# The Mediation of Circulating Inflammatory Proteins in the Causal Pathway From Immune Cells to Delirium

**DOI:** 10.1002/brb3.70811

**Published:** 2025-09-01

**Authors:** Tongshuai Liu, Xia Bi, Fuguo Ma, Yanlin Chen

**Affiliations:** ^1^ Department of Anesthesiology Qingdao Municipal Hospital Qingdao Shandong China; ^2^ Department of Anesthesiology The Affiliated Hospital of Qingdao University Qingdao Shandong China

**Keywords:** circulating inflammatory proteins, delirium, immune cells, mediation analysis, Mendelian randomization

## Abstract

**Objective:**

Observational research indicates that immune cells, in conjunction with circulating inflammatory proteins, could serve two functions in the development of delirium, despite the fact that the exact mechanisms remain ambiguous. The objective of this research is to identify the specific pathways through which immune cells trigger delirium and to evaluate the possible function of circulating inflammatory proteins as intermediaries.

**Methods:**

A two‐sample Mendelian randomization (MR) analysis was conducted using summary‐level data from genome‐wide association studies, which included 731 immune cells, 91 inflammatory proteins, and their association with delirium. The causal relationships between immune cells, inflammatory proteins, and delirium were systematically assessed through multivariable Mendelian randomization (multivariable MR). Following this, sensitivity analyses were performed using the Cochran *Q* test to evaluate heterogeneity, alongside MR‐Egger tests to examine pleiotropy. Finally, a two‐step methodology was employed to identify the inflammatory proteins that mediate the impact of immune cells on the onset of delirium.

**Results:**

Multivariable MR revealed 14 immune cell types that exhibited a causal relationship with delirium, along with 4 inflammatory proteins associated with this condition. Through a two‐step analysis, it was determined that 2 inflammatory proteins mediate the influence of 2 immune cell phenotypes on delirium. Specifically, CD25 on IgD^‐^ CD_38_dim is linked to the inflammatory protein LIGHT, which accounts for a mediation percentage of 9.65%. Meanwhile, CD127 on CD45RA^‐^ CD4, not Treg, is associated with the inflammatory protein CCL20, showing a mediation percentage of 9.78%.

**Conclusion:**

This study conducted a thorough investigation into the causal relationships between immune cells and delirium, while also assessing the role of inflammatory proteins as mediators. The results aid in recognizing individuals who are at an elevated risk for experiencing delirium while also offering novel perspectives for proactive preventive measures and therapeutic strategies within clinical environments.

## Graphical abstract

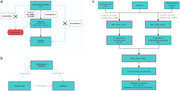

## 1 Introduction

Delirium is a prevalent neuropsychiatric syndrome marked by the abrupt onset of temporary disturbances in consciousness and attention, accompanied by a comprehensive disruption of cognitive and behavioral functions. Primarily affecting the elderly, its reported incidence varies depending on the population and context in which it is studied, and it is linked to adverse consequences in both the short term and the long term (McCusker et al. 2001). Numerous clinical studies have established a link between delirium and increased mortality, prolonged cognitive impairments, higher rates of complications, greater economic burdens, and an intensified strain on healthcare systems and caregivers (Chen et al. 2025; Thom et al. 2019). Unfortunately, delirium often goes underdiagnosed and undertreated. Thus, early detection and management are essential for enhancing patient outcomes.

Delirium can be viewed as an “acute brain failure,” a multifactorial syndrome comparable to acute heart failure. It emerges acutely in response to harmful stimuli (such as major surgery or sepsis) and may indicate the capacity of cognitive reserve (Brummel et al. 2024; Li et al. 2024), reflecting the brain's resilience against external challenges. In this context, delirium may represent a decline in fragile cerebral reserve capacity (Inouye et al. 2014). Although the pathophysiological mechanisms of delirium remain unclear, it is widely accepted that a multitude of biological factors contribute to this phenomenon, encompassing inflammatory responses, metabolic conditions, neurotransmitters, physiological stressors, electrolyte imbalances, and genetic components. These factors disrupt neuronal networks by directly or indirectly affecting the activity of neurons and glial cells (Ormseth et al. 2023). The emergence of delirium involves complex and dynamic interactions among several risk factors. Numerous disorders linked to delirium exhibit the activation of an inflammatory cascade, which is often accompanied by a rapid release of inflammatory mediators. Acute peripheral inflammatory stimuli can trigger the activation of brain parenchymal cells, the production of pro‐inflammatory cytokines, and the generation of inflammatory mediators within the central nervous system. These neuroinflammatory changes can lead to neuronal and synaptic dysfunction, resulting in subsequent neurological and cognitive impairments (Cerejeira et al. 2010).

It is widely believed that there is no close connection between the immune system and the nervous system, a concept referred to as central nervous system immune privilege. Nonetheless, recent investigations have uncovered that under certain conditions, pathological interactions between the immune and nervous systems can occur, potentially resulting in neurological or psychiatric disorders (Rustenhoven and Kipnis 2022). For instance, a recent Mendelian randomization (MR) study revealed a causal relationship between immune cell proteins and the onset of Parkinson's disease (Li et al. 2025). Immune cells can infiltrate brain parenchyma through the blood‐brain barrier and cerebrospinal fluid (Engelhardt and Ransohoff 2012; Hickey 1991), and recent findings have identified an extensive network of brain lymphatic drainage from the dura mater, in conjunction with the vascular routes that link cranial bone marrow to the surface of the brain (Da Mesquita et al. 2018; Herisson et al. 2018). This indicates that the brain is an integral part of an immune surveillance network that spans the entire body. The intricate relationships that exist between the immune system and the brain are fundamental to understanding the development of diseases affecting the central nervous system. For example, CD4 T cells are well recognized in neurology as key mediators in autoimmune diseases, such as the function of CD4 T cells in oligodendrocytes during multiple sclerosis (Komuczki et al. 2019). In addition to their role in autoimmune disorders, CD4 T cells have also been associated with neurodegenerative diseases, including Parkinson's disease (Roodveldt et al. 2024), Alzheimer's disease (Sirkis et al. 2024), and stroke (Wang et al. 2024). Although there is a growing recognition of the contribution of immune cells to neuroinflammation and neurodegenerative mechanisms (Castro‐Gomez and Heneka 2024; Jin et al. 2024; Zhou et al. 2017), their precise involvement in the onset of delirium is still not well understood.

Based on genetic instrumental variables derived from genome‐wide association studies (GWAS), MR offers methodological innovations for causal inference in epidemiological research. The methodological foundation of this approach lies in the random distribution of genetic variations during gamete formation, which allows for the construction of genetic proxy variables to estimate the causal effects between exposure factors and clinical outcomes. Compared to traditional observational studies, this genetic instrumental variable method effectively mitigates the false associations caused by environmental confounding factors (Ference et al. 2021). Consequently, our objective is to employ MR analysis to elucidate the causal connections between immune cells and the incidence of delirium, while also examining the degree to which circulating inflammatory proteins contribute to this relationship.

## 2 Materials and Methods

### 2.1 Study Design

**FIGURE 1:**
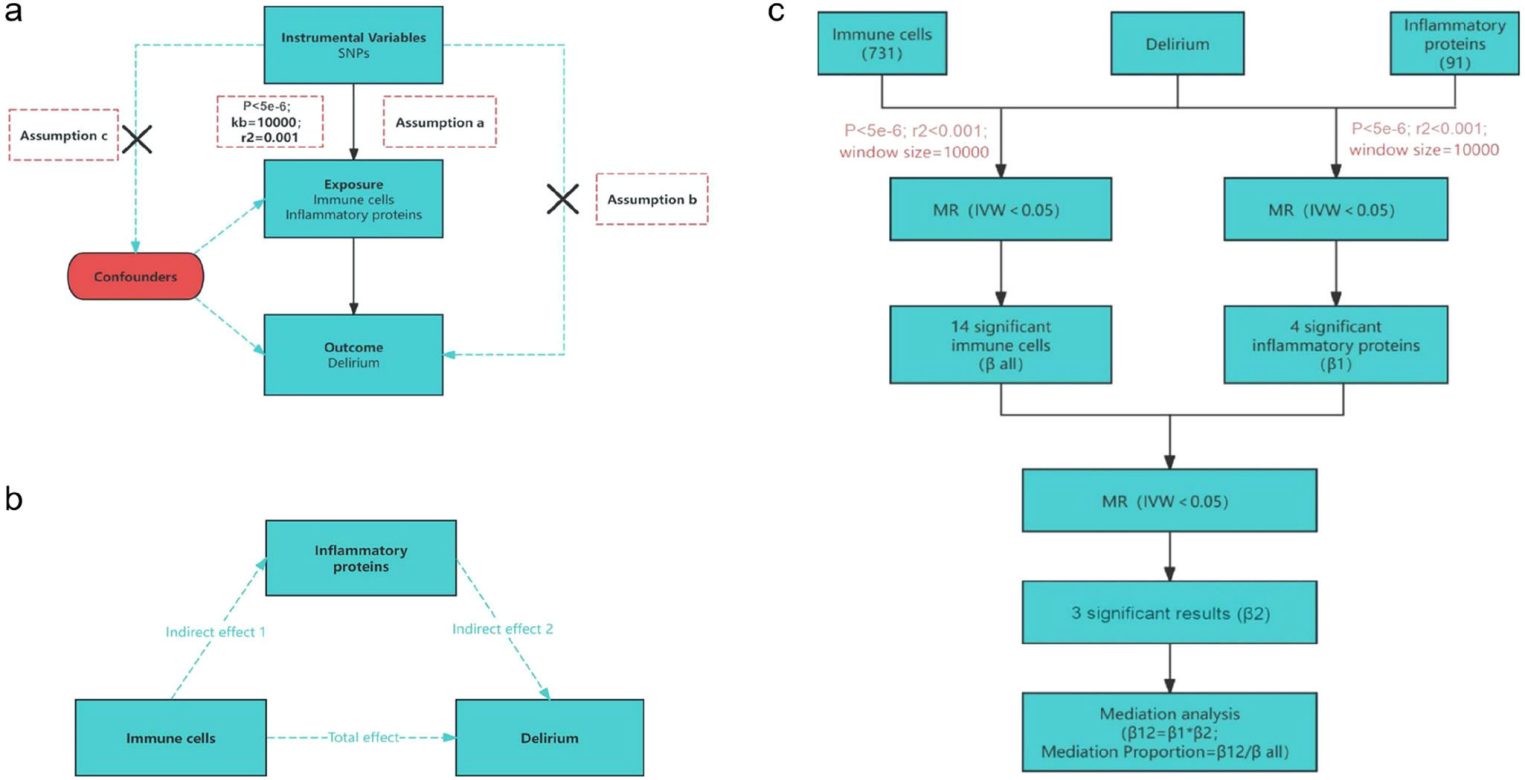
Study design and MR framework. Overview of the two‐sample MR and multivariable MR framework used to explore the causal pathway from immune cells to delirium via circulating inflammatory proteins. **(a)** Instrumental variable assumptions for MR, **(b)** Schematic of immune cells → inflammatory proteins → delirium mediation pathway, and **(c)** Multistage MR model workflow. **Abbreviations**: MR, Mendelian randomization; IV, instrumental variable; GWAS, genome‐wide association study; SNP, single‐nucleotide polymorphism.

In MR analysis, inflammatory proteins and their relationship to delirium are examined using single‐nucleotide polymorphisms (SNPs) that are strongly linked to these exposures, which are commonly selected as instrumental variables (IVs). To ensure the effectiveness of the MR analysis, the IVs must satisfy the following three key assumptions (Figure 1a): (1) Strong association assumption: The IV should have a strong association with the exposure factors, guaranteeing that these genetic variations sufficiently account for the differences observed in the exposure. (2) Independence assumption: In order to reduce bias, it is essential that the IV remain uncorrelated with confounding variables that affect both the exposure and the resultant outcome. (3) Exclusivity assumption: The IV must influence the outcome exclusively via the exposure factor, ensuring that no additional direct pathways are involved in affecting the outcome (Emdin et al. 2017).

In the realm of causal analysis, the influence of exposure variables on resultant outcomes can be divided into two distinct categories: total effects and indirect effects. Total effects denote the direct influence that exposure variables exert on the outcome, whereas indirect effects arise when the exposure impacts the outcome through intermediary factors. To investigate this relationship, we utilize two‐sample MR and multivariable MR methodologies. In this study, immune cells are designated as the exposure variable, while delirium is identified as the outcome variable. Based on the hypothesis of systemic inflammation, we prioritize the inclusion of peripheral inflammatory mediators within the causal mediation analysis framework. By constructing a multistage mediation MR model, this study design aims to elucidate the cascading causal pathway of immune cells → inflammatory mediators → delirium, while quantifying the regulatory efficacy coefficients (β_mediation) of specific chemokines/cytokines within the neuroimmune axis. (Figure 1b).

In this study, we focus on examining the causal connections between immune cells and delirium, along with the interactions involving inflammatory proteins. Initially, we explore how changes in immune cell levels might contribute to the development of delirium, seeking to identify the potential role of the immune system in this process. Following this, we investigate the relationship between inflammatory proteins and delirium, analyzing their function within the nervous system and their significance to the pathological mechanisms behind delirium. Last, we assess the complex interaction between immune cells and inflammatory proteins to reveal how they work together to form a network that drives the pathological progression. By applying the coefficient product method, we can quantitatively evaluate the indirect influence of these exposure factors on delirium outcomes, confirming the mediating effect of inflammatory proteins in the relationship between immune cells and delirium and clarifying their specific role in the overall causal framework. A detailed depiction of the research design is shown in Figure 1c.

### 2.2 Data Sources

The immunogenetic information employed in the present investigation is derived from a GWAS conducted in 2020, which encompassed 3757 participants of European descent. This study specifically examined 272 variables associated with blood immune cells, incorporating a total of 731 traits pertinent to the immune system (Orrù et al. 2020). The entire dataset is accessible in the GWAS catalog database, specifically under the login IDs ranging from GCST90001391 to GCST90002121, which can be located at https://www.ebi.ac.uk/gwas/.

The genetic information of circulating inflammatory proteins is derived from a genome‐wide protein quantitative trait locus (pQTL) study, which utilized the Olink Target platform to analyze 91 proteins in the plasma of 14,824 participants, representing 97.4% of the European population (Zhao et al. 2023). The complete dataset is available for free in the GWAS catalog database, accessible by logging in with IDs GCST90274758 to GCST90274848.

The genetic data on delirium is sourced from the FinnGen database, which includes 2,612 cases and 325,306 controls (100% of European ancestry) (https://www.finngen.fi/en). The definition of delirium is based on the International Classification of Diseases, 10th Edition (ICD‐10).

### 2.3 Instrumental Variable Selection

In the present investigation, we commenced by identifying SNPs linked to immune cells, inflammatory proteins, and delirium through a genome‐wide association study, using these SNPs as instrumental variables in the MR analysis. To validate the initial assumption of MR, which posits a robust correlation between the IV and the exposure variable, we established a stringent threshold of *p* < 1×10^^−8^ for SNP screening. Nonetheless, this rigorous criterion yielded a markedly limited number of instrumental variables. To address this limitation and to ensure an ample representation of positive SNPs that would bolster the statistical power of our analysis, we subsequently relaxed the *p*‐value threshold to *p* < 5×10^^−6^. This adjustment facilitated the acquisition of a broader array of genetic markers pertinent to the exposure variable. However, it is essential to acknowledge that genetic variations located in proximity on the genome tend to be inherited together, thereby increasing the likelihood of their co‐occurrence on the same chromosome relative to random chance. Consequently, we employed the clump_data() function from the R package TwoSampleMR to impose constraints on linkage disequilibrium parameters (with a kilobase pairs (kb) setting of 10,000 and an *r* ^2^ value of 0.001), thereby ensuring the independence of the selected SNPs.

To improve dataset reliability, the harmonize_data() function was used to remove potential duplicates or palindromic sequences, with the parameter set to “action = 2.” Next, the *F* statistic for each SNP was calculated using the formula F = (N‐K‐1)*R* ^2^/K(1‐*R* ^2^), which assesses the strength of the association between genetic loci and exposure factors. Following standard guidelines, an *F* value below 10 signifies that the SNP functions as a weak instrumental variable, leading to its exclusion from further analysis. In this formula, *R* ^2^ represents the proportion of variance attributable to the IV, *N* denotes the total sample size, and K stands for the number of SNPs. This thorough screening and validation process ensured that the selected instrumental variables maintained strong statistical independence and validity for the subsequent MR analysis.

### 2.4 Statistical Analysis

We employed the multivariable MR approach to analyze the potential associations between immune cells, inflammatory proteins, and delirium in a pairwise manner. This method effectively assesses the impact of multiple exposure factors on the outcome variable, minimizing the influence of confounding variables. Utilizing the R 4.4.2 environment, we conducted our specific analyses through the R package TwoSampleMR, ensuring the rigor and validity of our analytical processes. In assessing causal relationships, we employed five different methods: IVW, MR‐Egger regression, weighted median, weighted mode, and simple mode. IVW was selected as the main method for analysis because of its ability to account for heterogeneity in causal estimates. By utilizing meta‐analytic techniques for ratio estimation, IVW provides unbiased results, assuming that all chosen instrumental variables are valid (Verbanck et al. 2018). To ensure the reliability of the causal relationships and to mitigate the influence of critical factors that could potentially distort the causal assumptions, we instituted several quality control measures aimed at evaluating the sensitivity, heterogeneity, and multiplicity of the study outcomes. Initially, we utilized the IVW method to compute the *p‐*value corresponding to the Cochran *Q* test, which serves as an evaluation of heterogeneity's effects (Hemani et al. 2018). Subsequently, we employed MR‐Egger intercept correction, aimed at addressing pleiotropy and eliminating outliers (Bowden et al. 2016). A *p‐*value exceeding 0.05 suggests that both heterogeneity and pleiotropy do not substantially affect the findings of the study. Ultimately, we performed a leave‐one‐out sensitivity analysis to ascertain whether any specific SNP had a notable impact on the MR results.

To assess the mediating role of inflammatory proteins, we first applied the IVW method to calculate the total effect (β_all) between immune cells and delirium. Next, we determined the mediating effect (β1β2) by calculating the product of the β coefficient that represents the association between inflammatory proteins and delirium (β1) and the β coefficient reflecting the connection between immune cells and inflammatory proteins (β2). Finally, we defined the mediation proportion as β1β2/β_all, which serves as a measure for evaluating the mediating function of inflammatory proteins within the causal pathway linking immune cells to delirium (Bowden et al. 2016).

## 3 Results

### 3.1 Selection of IVs

Based on the established screening criteria (*p* < 5×10^^−6^, kb = 10,000, *r* ^2^ = 0.001), we identified 144 independent SNPs linked to 15 immune cells associated with delirium, along with 86 SNPs related to 4 inflammatory proteins in the same condition. Furthermore, all IVs had an *F*‐statistic exceeding 10, which minimizes the risk of weak instrument bias. Comprehensive details regarding each IV can be found in Supplementary Table.

### 3.2 Causal Effects of Immune Cells on Delirium

**FIGURE 2:**
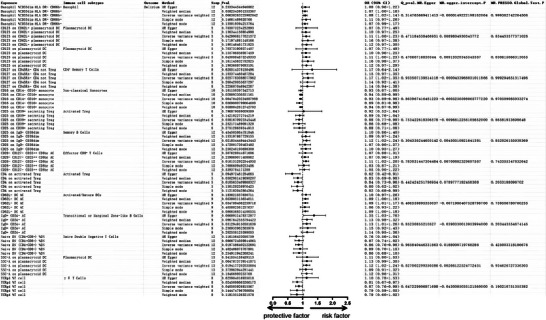
Forest plot of causal associations between 14 immune cell phenotypes and delirium using five MR methods. The plot shows the ORs and 95% CIs for 14 immune cell phenotypes that demonstrated significant causal associations with delirium in multivariable MR analysis. Results were obtained using five MR methods: IVW, MR‐Egger, weighted median, weighted mode, and simple mode. The x‐axis represents the OR on a logarithmic scale, and the y‐axis lists the 14 immune cell phenotypes. OR > 1 indicates increased risk of delirium, whereas OR < 1 indicates a potential protective effect. **Abbreviations**: CI, confidence interval; IVW, inverse variance weighting; MR, Mendelian randomization; OR, odds ratio; SNP, single‐nucleotide polymorphism.

In the analysis utilizing multivariable MR, the IVW method was employed as the main analytical approach. This indicates that there may be a causal association between 15 specific immune cell types and the occurrence of delirium. (Supplementary Table). To account for potential confounding factors, we performed sensitivity analyses on the 15 causal associations and discovered that the immune cell phenotype CD39^+^ resting Treg % CD4 Treg exhibited multifunctionality (*p* < 0.05). This finding implies that the exposure variable could influence the outcome through various biological pathways or in different ways depending on the environmental context. After excluding this particular factor, 14 immune cells continued to show a significant causal impact on delirium (Figure 2).

**FIGURE 3:**
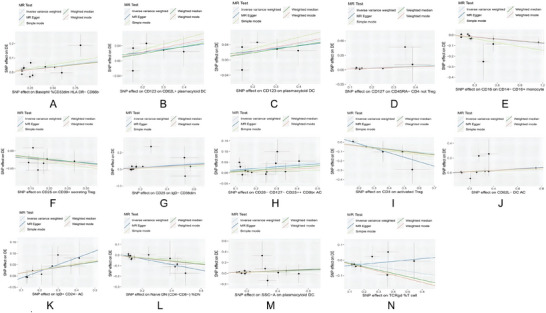
Scatter plots of genetic associations between 14 immune cell phenotypes and delirium using five MR methods. Panels A–N correspond to the 14 immune cell phenotypes that showed significant causal associations with delirium in multivariable MR analysis. Each point represents the effect of an SNP on the immune cell phenotype (x‐axis) and on delirium (y‐axis). Colored regression lines represent causal estimates from five MR methods: IVW, MR‐Egger, weighted median, weighted mode, and simple mode. The x‐axis shows the SNP effect on the immune cell phenotype, and the y‐axis shows the SNP effect on delirium. A positive slope indicates increased risk, and a negative slope indicates a potential protective effect. *Note*: Delirium is referred to as “DE” in the figure. **Abbreviations**: IVW, inverse variance weighting; MR, Mendelian randomization; SNP, single‐nucleotide polymorphism.

Among the immune cells studied, 9, including CD123 on CD62L^+^ plasmacytoid DC, CD123 on plasmacytoid DC, and CD127 on CD45RA^−^ CD4^+^ not Treg cells, showed a potential positive correlation with delirium (OR > 1). Conversely, 5 immune cells—namely, CD16 on CD14‐CD16^+^ monocytes, CD25 on CD39^+^ secreting Treg cells, CD39^+^ resting Treg cells % CD4 Treg cells, CD4 on activated Treg cells and TCRgd % T cells—were found to negatively regulate the risk of developing delirium (OR < 1). Figure 3 is a scatter plot of the MR analysis between 14 immune cell types and delirium.

**FIGURE 4:**
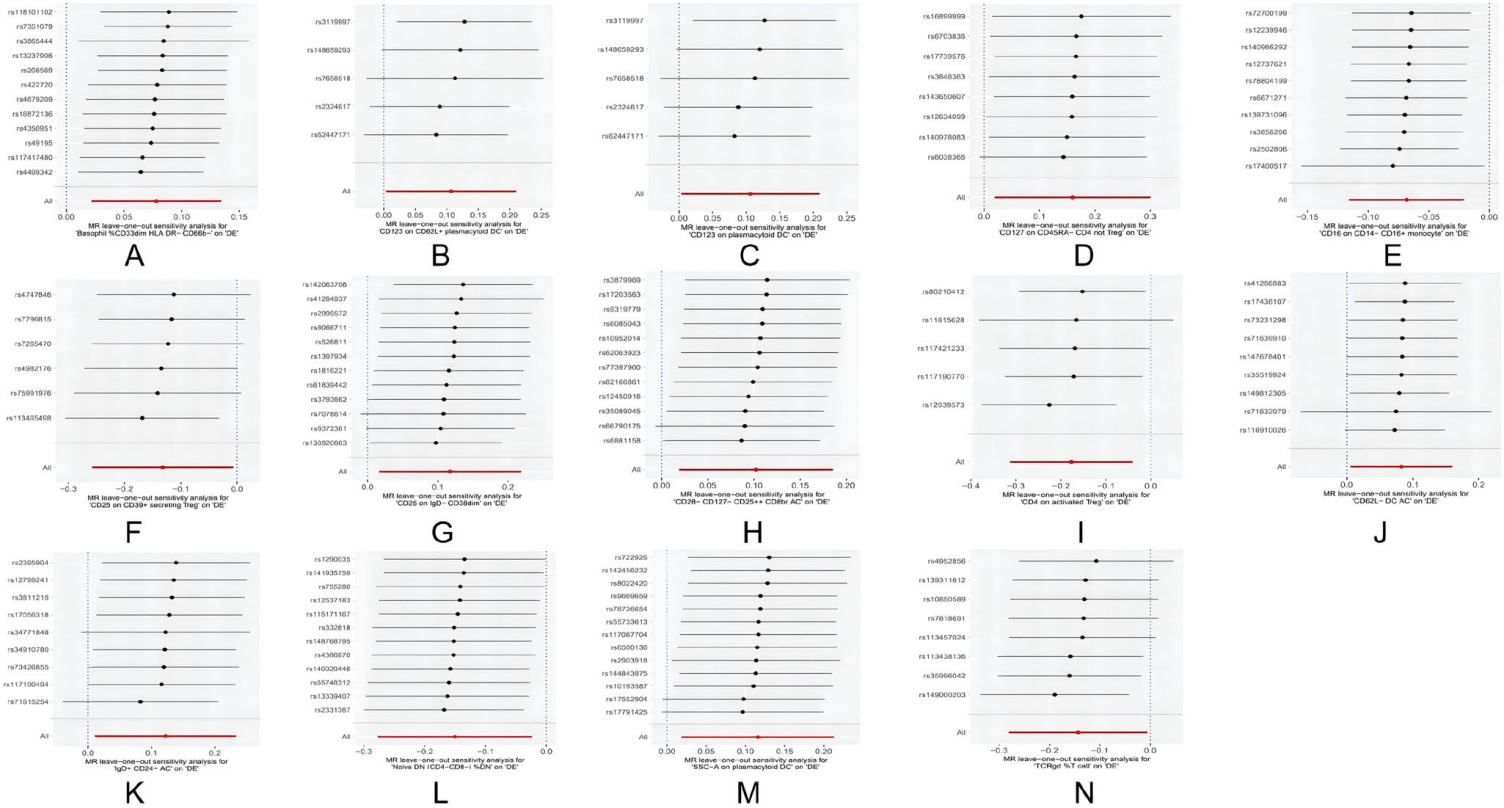
Leave‐one‐out sensitivity analyses of 14 immune cell phenotypes in multivariable MR. Panels A–N display leave‐one‐out analysis results using the IVW method. Each dot represents the causal estimate (β) after sequentially removing one SNP. Black dots indicate *β* values with 95% confidence intervals, and red dots denote statistically significant effects. The x‐axis represents the causal effect size (*β*) on delirium, and the y‐axis corresponds to each excluded SNP (rsID). The results indicate that no single SNP had a disproportionate influence on the MR estimates, supporting the robustness of the causal inference. All SNPs used as instrumental variables were selected at a genome‐wide significance threshold of *p* < 5 × 10^^‐6^. **Abbreviations**: IVW, inverse variance weighting; MR, Mendelian randomization; SNP, single‐nucleotide polymorphism.

Subsequently, we performed a leave‐one‐out analysis focusing on the previously mentioned 14 immune cell phenotypes, as shown in Figure 4. This analysis entailed the systematic omission of each SNP in turn while assessing the effect size of the remaining SNPs. The findings revealed that there were no notable differences in the effect sizes of the 14 immune cell phenotypes when comparing the data prior to and following the exclusion, indicating that no individual SNP exerts a substantial influence on the MR estimates.

### 3.3 Causal Effects of Circulating Inflammatory Proteins on Delirium

**FIGURE 5:**
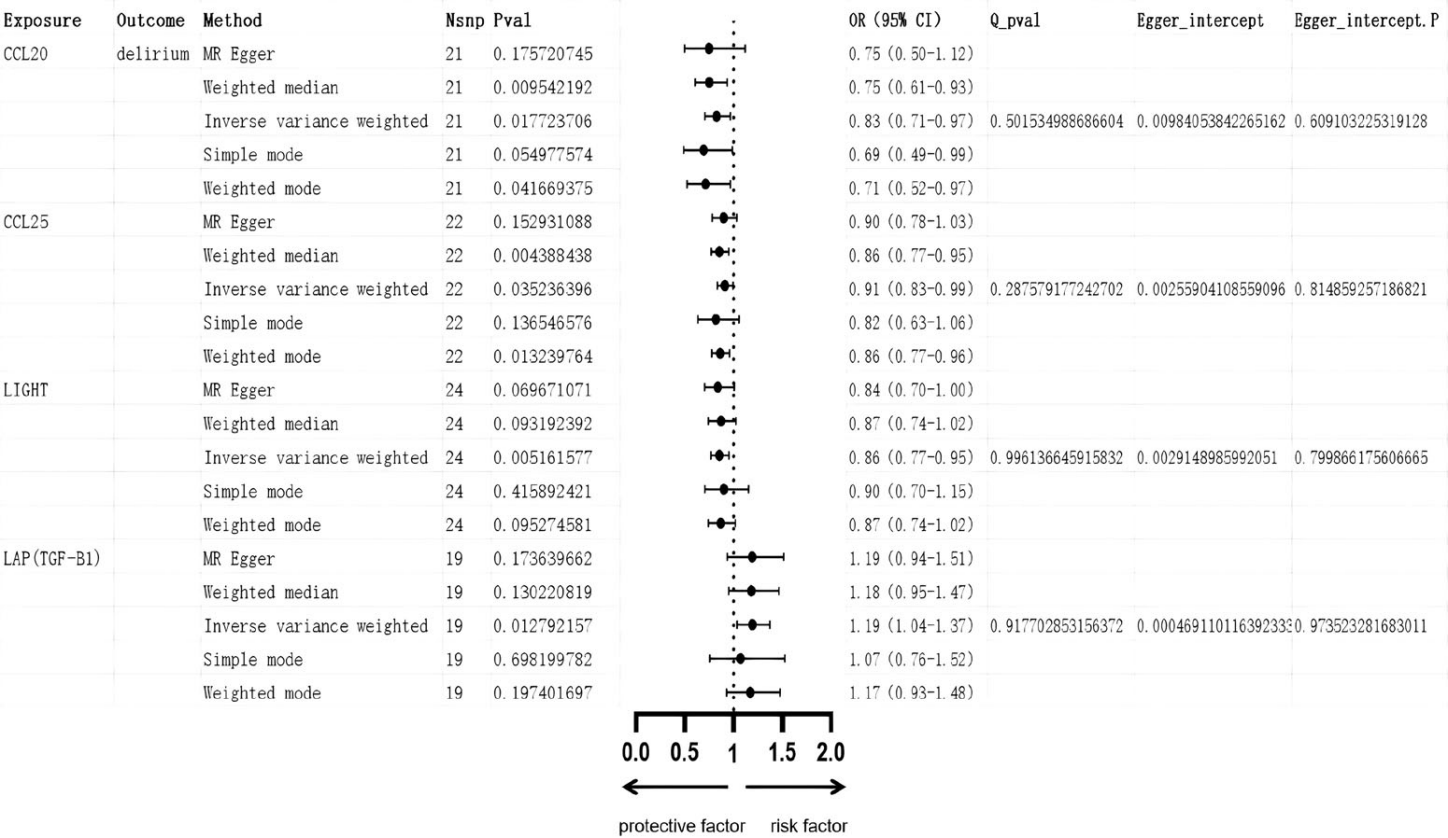
Forest plot of causal associations between four circulating inflammatory proteins and delirium using five MR methods. The plot displays the ORs and 95% CIs for four circulating inflammatory proteins that were significantly associated with delirium in multivariable MR analysis. Results were obtained using five MR methods: IVW, MR‐Egger, weighted median, weighted mode, and simple mode. The x‐axis represents the OR on a logarithmic scale, and the y‐axis lists the four inflammatory proteins (CCL20, CCL25, LIGHT/TNFSF14, LAP/TGF‐β1). OR > 1 indicates increased risk of delirium, whereas OR < 1 suggests a potential protective effect. **Abbreviations**: CI, confidence interval; IVW, inverse variance weighting; MR, Mendelian randomization; OR, odds ratio; SNP, single‐nucleotide polymorphism.

Consistent with the above methods, we utilized the multivariable MR approach to identify four inflammatory proteins that have a causal impact on delirium (Supplementary Table). In our findings, we observed that elevated levels of CCL20, CCL25, and LIGHT were inversely associated with the likelihood of experiencing delirium. Conversely, LAP (TGF‐B1) showed a positive regulatory influence on the incidence of delirium. Further sensitivity analyses revealed that these four proteins did not display substantial heterogeneity or horizontal pleiotropy in relation to their causal impacts on delirium. (*p* > 0.05) (Figure 5).

**FIGURE 6:**
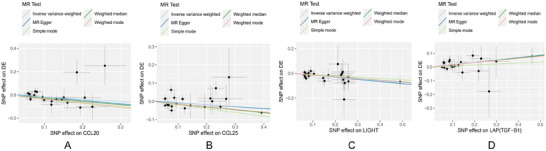
Scatter plots of genetic associations between four circulating inflammatory proteins and delirium. Panels A–D correspond to four inflammatory proteins significantly associated with delirium in multivariable MR analysis. Each point shows the genetic effect of a single SNP on both the inflammatory protein (x‐axis) and delirium (y‐axis). Colored regression lines indicate causal estimates from five MR methods: IVW, MR‐Egger, weighted median, weighted mode, and simple mode. Positive slopes indicate increased risk, while negative slopes suggest a potential protective effect. **Abbreviations**: IVW, inverse variance weighting; MR, Mendelian randomization; SNP, single‐nucleotide polymorphism.

The scatter plot of the MR analysis between four inflammatory proteins and delirium is shown in Figure 6.

**FIGURE 7:**
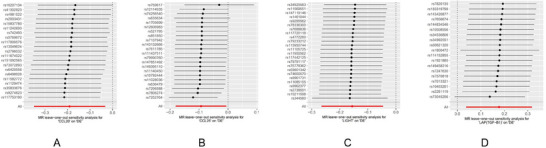
Leave‐one‐out sensitivity analysis of four circulating inflammatory proteins in multivariable MR. Panels A–D display leave‐one‐out analysis results using the IVW method. Each dot represents the causal estimate (*β*) after sequentially removing one SNP. Black dots indicate β values with 95% confidence intervals, and red dots denote statistically significant effects. The x‐axis shows the causal effect size (β) on delirium, and the y‐axis lists the excluded SNPs (rsID). The analysis demonstrates that no individual SNP exerted a disproportionate influence on the MR estimates, indicating the robustness of the causal inference. All SNPs used as instrumental variables were selected at a genome‐wide significance threshold of *p* < 5×10^−6^. **Abbreviations**: β, effect size; CI, confidence interval; IVW, inverse variance weighting; MR, Mendelian randomization; SNP, single‐nucleotide polymorphism.

Figure 7 shows the leave‐one‐out analysis conducted on four inflammatory proteins, which revealed that there were no notable variations in effect sizes when each protein was excluded sequentially. This observation suggests that no individual SNP exerted a substantial influence on the MR estimates.

### 3.4 Mediated MR Analysis

**FIGURE 8:**
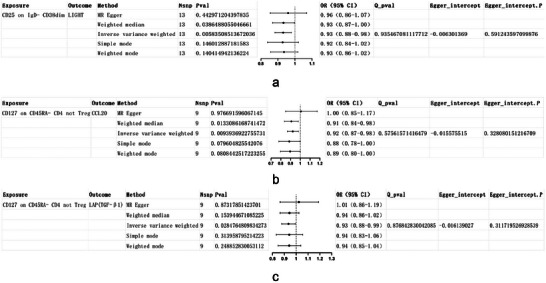
Forest plots of causal associations between two immune cell phenotypes and three circulating inflammatory proteins using MR methods. Panels a–c illustrate the causal relationships analyzed using multiple MR methods: **(a)** CD25 on IgD^−^ CD38dim on LIGHT (TNFSF14); **(b)** CD127 on CD45RA^−^ CD4⁺ non‐Treg on CCL20; and **(c)** CD127 on CD45RA^−^ CD4⁺ non‐Treg on LAP (TGF‐β1). Each forest plot shows the ORs with 95% CIs obtained from MR‐Egger, weighted median, inverse variance weighted, simple mode, and weighted mode analyses. The x‐axis represents the OR, and the y‐axis lists the MR methods. OR > 1 indicates increased risk of delirium, whereas OR < 1 suggests a potential protective effect. **Abbreviations**: CI, confidence interval; MR, Mendelian randomization; OR, odds ratio; SNP, single‐nucleotide polymorphism.

Building upon the immune cells and circulating inflammatory proteins previously recognized to have a substantial causal link with delirium, we undertook an additional analysis utilizing the multivariable MR approach. In this analysis, immune cells were regarded as the exposure and inflammatory proteins as the outcome to elucidate their interrelationship. Consequently, we uncovered three causal associations between two distinct immune cell phenotypes and three inflammatory proteins. Furthermore, we executed a straightforward sensitivity analysis on the MR findings to reinforce their reliability. As illustrated in Figure 8, the outcomes demonstrated no significant heterogeneity or pleiotropy.

**FIGURE 9:**
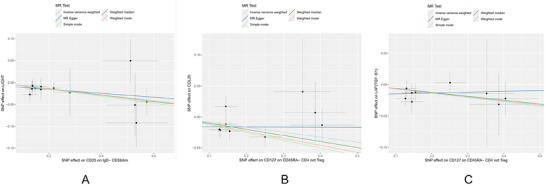
Scatter plots of causal associations between immune cell phenotypes and their corresponding inflammatory protein mediators. Each panel illustrates the relationship between a single immune cell phenotype and its corresponding inflammatory protein mediator in the mediation MR analysis. Each point represents the effect of an SNP on the immune cell phenotype (x‐axis) and on the inflammatory protein (y‐axis). The x‐axis shows the SNP effect on the immune cell phenotype, and the y‐axis shows the SNP effect on the protein mediator. A positive slope indicates a positive causal correlation, meaning higher immune cell levels are associated with higher levels of the corresponding protein; a negative slope indicates a negative causal correlation, meaning higher immune cell levels are associated with lower protein levels. **Abbreviations**: IVW, inverse variance weighting; MR, Mendelian randomization; SNP, single‐nucleotide polymorphism.

The scatter plot of the MR analysis between two immune cell phenotypes and three inflammatory proteins is shown in Figure 9.

**FIGURE 10:**
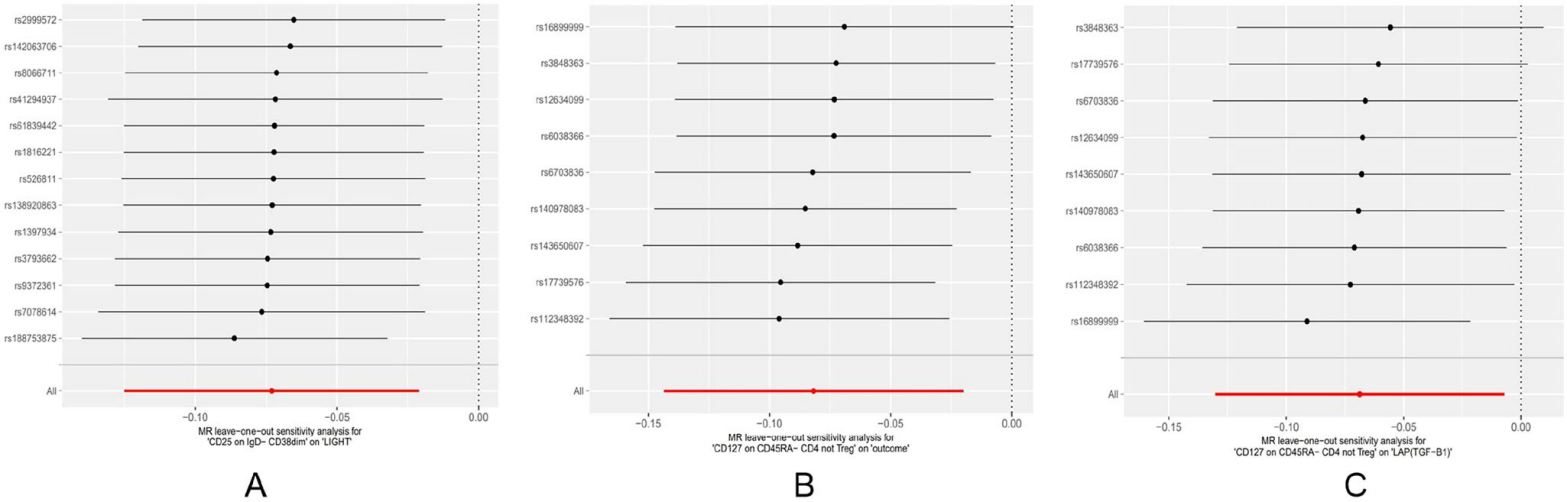
Leave‐one‐out sensitivity analyses of immune cell–protein pairs in multivariable MR. Panels A–C show leave‐one‐out analysis results using the IVW method. Each dot represents the causal estimate (β) after removing one SNP; black dots indicate *β* values with 95% confidence intervals, and red dots mark significant effects. The x‐axis shows the causal effect size (*β*), and the y‐axis lists the excluded SNPs (rsID). Panel A shows CD25 on IgD^−^ CD38^dim as the exposure and LIGHT as the outcome. Panel B presents CD127 on CD45RA^−^ CD4 not Treg as the exposure and CCL20 as the outcome. Panel C presents the same exposure (CD127 on CD45RA^−^ CD4 not Treg) but with LAP (TGF‐β1) as the outcome. No individual SNP had a disproportionate influence on the MR estimates, indicating robust causal inference. All SNPs used as instrumental variables were selected at genome‐wide significance threshold of *p* < 5 × 10^^−6^. **Abbreviations**: IVW, inverse variance weighting; MR, Mendelian randomization; SNP, single‐nucleotide polymorphism.

The outcomes of the leave‐one‐out analysis concerning two immune cell phenotypes and three inflammatory proteins reveal that there are no notable differences in effect sizes prior to and following the exclusion process. This suggests that no individual SNP exerts a significant influence on the MR estimates (Figure 10).

### 3.5 Potential Mediation Effect Calculation

Within the framework of mediation analysis, a crucial criterion for inflammatory proteins to function as mediators is that the indirect effect of immune cells on delirium, mediated through these proteins, should align in direction with the total impact of immune cells on delirium. Consequently, upon elucidating the interactions between two unique immune cell phenotypes, three inflammatory proteins, and the occurrence of delirium, we proceeded to compute β_all (representing the influence of immune cells on delirium), β1 (the influence of immune cells on inflammatory proteins), and β2 (the influence of inflammatory proteins on delirium). Following the determination of the indirect effect (β1β2), we assessed the percentage of the mediation effect by dividing this indirect effect by the total effect. The results are illustrated in the accompanying table, which indicates that two inflammatory proteins served as mediators in the connection between the two immune cell phenotypes and delirium. Notably, the mediation percentage for LIGHT was found to be 9.65%, whereas for CCL20, it was recorded at 9.78%.

The mediation proportion of inflammatory proteins in the causal relationship between immune cells and delirium.

**Table:** 

Exposure	Outcome	β_all	β1	β2	Mediation proportion	*p*
CD25 on IgD^−^ CD38dim	LIGHT	0.118	−0.073	−0.156	9.65%	0.006
CD127 on CD45RA^−^ CD4 not Treg	CCL20	0.160	−0.082	−0.191	9.78%	0.009

*Note*: β_all = total effect of immune cell on delirium. β_1_ = effect of inflammatory protein on delirium. β_2_ = effect of immune cell on inflammatory protein. Mediation proportion = (β_1_ × β_2_) / β_all × 100%. Analysis was conducted using the IVW method; all SNPs were selected with *p* < 5 × 10^^−6^.

## 4 Discussion

This study utilized the MR methodology to explore the potential causal relationships between inflammatory proteins present in circulation and various immune cell phenotypes, particularly within the framework of delirium. Our findings identified two noteworthy associations where immune cell phenotypes were influenced by circulating inflammatory proteins, thereby affecting the likelihood of developing delirium. These findings provide fresh insights into the fundamental processes associated with delirium and could aid in uncovering new treatment options.

Our research is the first to establish a causal connection between immune cell phenotypes and delirium, aligning with existing studies. Our findings indicate that 14 distinct types of immune cell phenotypes serve as risk factors for the development of delirium. This includes six types of T cell phenotypes, four types of dendritic cell (DC) phenotypes, two types of B cell phenotypes, one type of granulocyte phenotype, and one type of monocyte phenotype. Previous research has demonstrated that the activation of T cells leads to the release of cytokines such as IL‐6 and IL‐15, which are closely associated with the emergence of delirium (Gust et al. 2020). During the onset of delirium (Kunchok et al. 2019), abnormal activation or differentiation of T cells leads to immune responses in astrocytes, causing inflammation and damage in the nervous system, thereby triggering neurological symptoms in patients. Regulatory T cells (Tregs), a subtype of T cells, play a vital role in maintaining balance and self‐tolerance within the immune system. They are crucial for regulating immune responses, suppressing excessive immune reactions, and preventing autoimmune diseases (Cardenas et al. 2024). Dysfunction or reduced numbers of Tregs can exacerbate neuroinflammation, subsequently increasing the risk of delirium (Cardenas et al. 2024). There is evidence suggesting that improving or restoring the function of Treg cells can alleviate postoperative cognitive dysfunction in aged mice (Zhou et al. 2023). During the inflammatory response in the brain, dendritic cells (DCs) recognize pathogens, ingest and process antigens, and present them to T cells via MHC molecules, thereby activating them. Simultaneously, DCs release pro‐inflammatory factors such as IL‐12, TNF‐α, and IL‐6, which attract other immune cells to the sites of inflammation. Activated DCs migrate to lymph nodes, amplifying inflammatory signals and enhancing specific immune responses (Worbs et al. 2017). Regarding B cells, some studies suggest that they may worsen neurocognitive impairment in neurodegenerative diseases, exhibiting significant pro‐inflammatory tendencies (Kim et al. 2021). An increase in B cells within the brains of Alzheimer's disease (AD) patients exacerbates AD‐related neuroinflammation by producing immunoglobulins and various pro‐inflammatory factors. The reduction or functional impairment of B cells within the brain facilitates the activation of microglia and leads to a decrease in the expression levels of TREM2, CLEC7A, and ITGAX in the hippocampus, thereby markedly attenuating the progression of AD (Kim et al. 2021). Research also indicates that granulocytes—including basophils, eosinophils, and neutrophils—contribute to the formation of extracellular traps (ETs) (Wu et al. 2021). Overexpression of ETs in the brain can compromise the integrity of the blood‐brain barrier (BBB) and promote neuroinflammation through the release of metalloproteinases, proteases, cytokines, extracellular histones, DNA, and ROS (Sun et al. 2024).As for monocytes, studies have reported that the percentage of CD14loCD16+ monocytes in ICU patients is regarded as an important immune marker for delirium, with higher levels observed in non‐survivors, correlating with poor prognosis (Lei et al. 2022). Monocytes, particularly microglia, play a significant role in the inflammatory response that follows sepsis, and their overactivation can lead to neurocognitive sequelae (Mein et al. 2023).

Recent investigations highlight that multiple elements instigating delirium activate inflammatory cascade mechanisms, which consequently result in the rapid discharge of inflammatory agents into the circulatory system. Compelling evidence points to the fact that acute peripheral inflammatory triggers provoke the activation of brain parenchymal cells, leading to the production of pro‐inflammatory cytokines and inflammatory substances within the central nervous system. Such alterations in neuroinflammation may precipitate neuronal and synaptic impairments, ultimately manifesting as behavioral and cognitive disturbances (Cerejeira et al. 2010). A recent study using MR also investigated the effects of circulating inflammatory proteins on various neurodegenerative disorders. The findings suggest that CD40 may exert neuroprotective effects in patients with multiple sclerosis through multiple cellular and molecular pathways (Gong et al. 2025). CCL20 is a C‐C chemokine that binds to the CCR6 chemokine receptor, promoting the migration of CCR6‐expressing immune cells to tissues. Its expression significantly increases during inflammation (Meitei et al. 2021). Increased concentrations of CCL20 in the chronic phase following ischemic brain injury can facilitate the infiltration and accumulation of Tregs, which suppress excessive immune responses by releasing inhibitory factors and directly inhibiting the function of other T cells (Ito et al. 2019). Research has shown that CCL20 significantly rises during remission in patients with bipolar disorder (BPD) receiving monotherapy, indicating its significant contribution throughout the duration of psychiatric conditions (Duan et al. 2022). Similarly, CCL25, also a C‐C chemokine with CCR9 as its specific ligand (Xu et al. 2020), exhibits elevated expression, which facilitates the migration of CD8^+^ T cells to infection sites in the brain, assisting in their spatial and temporal localization and thereby protecting the host from severe disease (Hilt et al. 2022). Studies have found that patients with post‐traumatic stress disorder (PTSD) show significantly reduced levels of CCL25, which correlates with the onset of PTSD, underscoring its potential significance in PTSD development (Zhang et al. 2020). LIGHT (lymphotoxin‐like inducible protein, also known as TNFSF14) is a cytokine belonging to the TNF superfamily, predominantly expressed in inflammatory effector cells. It activates endogenous signaling pathways by binding to herpesvirus entry mediator (HVEM) and lymphotoxin β receptor (LTβR), stimulating the activation of NF‐κB transcription factors, which subsequently modulate the gene expression associated with inflammatory responses, proliferation, and cell survival (Ware et al. 2022). In glioblastoma (GBM) patients, LIGHT targets tumor vasculature via its interaction with LTβR and HVEM, promoting vascular normalization, enhancing pericyte contractility, and rebuilding the endothelial barrier, thus improving vascular function and reducing tumor hypoxia. Moreover, LIGHT induces the formation of high endothelial venules (HEVs); through vascular normalization and improved blood perfusion, LIGHT may positively impact the survival, function, and repair of brain neurons (He et al. 2018). Research has demonstrated that LIGHT levels are significantly reduced in major depressive disorder (MDD), which may affect neuronal survival and cytokine production, highlighting its essential role in the pathophysiology of depression (Schmidt et al. 2019). Other studies have shown that LIGHT, as a plasma biomarker, is associated with the occurrence of delirium (Leung et al. 2024). LAP (Latency‐Associated Peptide) binds to TGF‐β1 (Transforming Growth Factor beta 1), maintaining TGF‐β1 in its inactive state. TGF‐β1 is a secreted signaling protein that regulates various cellular processes. LAP is a disulfide‐bonded dimer primarily responsible for keeping TGF‐β1 inactive, forming the so‐called latent TGF‐β (LTGF‐β) complex (Stachowski et al. 2020). Research has indicated that TGF‐β1 is a critical cytokine that can significantly inhibit the generation of superoxide (O_2_^−^) and nitric oxide (NO) in microglial cultures, providing neuroprotection. However, using LAP to neutralize the activity of TGF‐β1 can eliminate the inhibitory effect of hippocampal culture media on O_2_^−^ production, thus interfering with this neuroprotective mechanism. Consequently, overexpression of LAP (TGF‐β1) may lead to excessive activation of microglia and increased microglia‐mediated neurotoxicity (Herrera‐Molina and von Bernhardi 2005). Additionally, as a component of LAP (TGF‐β1), TGF‐β1 enhances the expression of agrin protein in neurons, which in turn supports synapse formation and aids in the clustering of acetylcholine receptors (AChR). LAP plays a crucial role in ensuring that TGF‐β1 is activated at the appropriate time, thereby promoting synapse formation and function (Feng and Ko 2008). Moreover, TGF‐β1 can encourage the differentiation of CD4^+^ T cells into Treg cells. When Treg cells lack TGF‐β1, they may become more sensitive to IL‐12, triggering autoimmune responses. Therefore, LAP (TGF‐β1) can reduce the conversion of CD4+ T cells to Treg cells and is essential for maintaining Treg cell stability and their inhibitory function (Choi et al. 2020).

Our mediation analysis revealed the role of two inflammatory proteins. First, with LIGHT mediating the inhibitory proportion of CD25 on IgD^−^ CD_38_dim cells in relation to delirium at a rate of 9.65%. Specifically, CD38 is a crucial cell surface glycoprotein involved in cell signaling and metabolic regulation, and it can be found on various immune cells, including B cells, T cells, and neutrophils (Piedra‐Quintero et al. 2020). The term “CD38dim” refers to a state where the expression of CD38 on the cell surface is low. IgD^−^ signifies the absence of IgD antibodies on B cells. CD25, also known as the IL‐2 receptor alpha chain (Peng et al. 2023), is a cell surface marker that helps identify specific subpopulations of B cells (Inaba et al. 2023). CD25 on IgD‐CD38dim describes a particular subset of B cells that lack IgD and exhibit low CD38 expression while still expressing CD25. This phenotype may indicate that these B cells are in a state of immune activation. On the other hand, LIGHT, as an integral component of the tumor necrosis factor superfamily, holds considerable importance in enhancing cerebral perfusion and contributes to the pathophysiology of depression, as previously mentioned. In the present investigation, we found that CD25 on IgD^−^ CD38dim was positively correlated with the occurrence of delirium, while it showed a negative correlation with LIGHT. Conversely, the expression of LIGHT was negatively associated with the incidence of delirium. This suggests that an increased expression of this immune cell phenotype may lead to delirium, potentially mediated by the suppression of LIGHT expression. Interestingly, our results suggest that CD25⁺ IgD^−^ CD38^dim B cells are specifically associated with the regulation of LIGHT (TNFSF14), but not other TNF superfamily members. While the precise mechanistic basis for this selectivity remains unclear, it is plausible that this B‐cell subtype, likely representing an activated or regulatory phenotype, has a unique transcriptional profile favoring LIGHT expression. Supporting this, single‐cell RNA sequencing data from the Human Protein Atlas indicate that TNFSF14 is preferentially expressed in B‐cell clusters within peripheral blood mononuclear cells (PBMCs), classified as “cell type enhanced” in this lineage (https://v23.proteinatlas.org/ENSG00000125735‐TNFSF14/single+cell+type). These findings provide preliminary biological support for the observed specificity. However, further validation using sorted subpopulations or single‐cell transcriptomic profiling of CD25⁺ B cells will be necessary to clarify the functional relevance of this interaction.

Second, our analysis revealed that CCL20 mediates the inhibitory proportion of CD127 on CD45RA^−^CD4 not Treg cells, regarding delirium at a rate of 9.78%. CD45RA‐CD4 not Treg represents a subset of non‐regulatory CD4 T cells, which are essential for the functioning of the immune system by activating, coordinating, regulating, and supervising both innate and adaptive immune responses (Raphael et al. 2020). CD4 T cell subsets can be classified into three primary categories: central memory T cells (TCM), effector memory T cells (TEM), and tissue‐resident memory T cells (TRM). Memory CD4 T cells are particularly significant in autoimmune diseases due to their longevity and effective responses to antigens. For example, in autoimmune diseases such as multiple sclerosis, a significant number of autoreactive memory CD4 T cells are identified.(Ostkamp et al. 2022) Experimental data demonstrate that autoreactive memory CD4 T cells are present during disease onset and directly impact pathologies (Balmas et al. 2023; Jelcic et al. 2018; Súkeníková et al. 2024). CD45 is a transmembrane glycoprotein that modulates the activities of both the innate and adaptive immune systems (Ahmed et al. 2023). CD45RA is a human variant of CD45 (Rheinländer et al. 2018), and its expression is silent in the context of CD127 on CD45RA^−^ CD4 not Treg cells. CD127, alternatively referred to as the interleukin‐7 receptor (IL‐7Rα) (Kaiser et al. 2023), is crucial for the development, survival, and differentiation of effector/memory CD4^+^ T cells. Studies have shown that knocking out the IL‐7Rα gene in CD4^+^ T cells results in a near‐complete disappearance of IL‐7Rα on the surface of these cells, leading to a significant decrease in their population (Azizi et al. 2024). Additionally, IL‐7Rα has been reported to impair IL‐2 receptor signaling and restrict the in vitro differentiation of Treg cells (Waickman et al. 2020). During immune responses, IL‐7Rα may indirectly influence the expression and function of CCL20 by enhancing the survival and functionality of T cells. Therefore, IL‐7Rα activity could affect CCL20 generation and its mediating immune responses (Loeffler et al. 2010). Further research indicates that IL‐7Rα influences the functionality of IL‐7, thereby regulating the activation of immune cell populations, while CCL20 is responsible for recruiting these activated immune cells (Yang et al. 2017). In our study, we found a positive correlation between CD127 on CD45RA^−^ CD4 not Treg cells, and the occurrence of delirium, whereas it had a negative correlation with CCL20. Additionally, the expression of CCL20 was negatively associated with delirium. This suggests that an increase in the expression of this immune cell phenotype may lead to the occurrence of delirium, potentially mediated by the suppression of CCL20 expression.

Although the mediation proportion identified in our study (∼9–10%) may appear modest, it is consistent with the polygenic nature of complex neuropsychiatric and neuroinflammatory traits. These conditions are typically shaped by a constellation of small‐effect genetic and immune‐related contributors, rather than by singular dominant mediators. Consistent with this, several recent MR studies have reported similarly modest mediation effects in brain–immune pathways. For instance, Xiao et al. (2025) found that circulating inflammatory proteins such as Artemin and LT‐α mediated between 5.6 and 7.8% of the effect from psoriasis to psychiatric traits, including broad depression and bipolar disorder. Similarly, in another multivariable MR study on epilepsy, microbial genera like Lachnospira and inflammatory proteins such as TNFSF11 exhibited comparable indirect effect proportions (6–10%) (Yang et al. 2024). Together, these findings underscore that even partial mediation (in the ∼5–10% range) is biologically plausible and consistent with expectations for complex traits. Furthermore, when such mediation involves potentially druggable targets—as is the case for LIGHT (TNFSF14) and CCL20 in our study—the results may be clinically meaningful and worthy of further translational investigation.

This research employed MR to investigate the causal link between immune cell activity and the occurrence of delirium, while adeptly adjusting for possible confounding variables. Through the utilization of pre‐existing extensive GWAS datasets, the research eliminated the requirement for extended follow‐up periods, consequently conserving both time and resources. Despite the relatively modest mediating influence of inflammatory proteins, our results underscore their importance within the context of the interplay between immune cells and delirium. We have identified specific immune cells and proteins that exhibit a causal relationship with delirium, thus providing essential perspectives for subsequent research endeavors. Despite the involvement of inflammatory proteins in mediating various biological processes in the connection between immune cells and delirium is somewhat constrained, their importance in immune reactions and the advancement of diseases must not be underestimated. A deeper examination of these inflammatory proteins may uncover possible therapeutic targets for clinical applications, facilitating the early identification and individualized treatment of delirium. Moving forward, a more comprehensive understanding of the causal interactions between immune cells and delirium, alongside the mediating roles of inflammatory proteins, may pave the way for innovative immunomodulatory therapies, subsequently improving the outlook and general life quality for those impacted by delirium.

This investigation is not without its significant limitations. Primarily, our analysis focuses exclusively on individuals of European descent, which could restrict the generalizability of our findings to other ethnic populations. Consequently, it is essential to incorporate a broader array of populations to achieve a thorough comprehension of the worldwide epidemiology of delirium, and future inquiries should strive to involve these varied cohorts. Secondly, to encompass a greater number of phenotype‐associated genetic variants, we adopted a more lenient criterion for selecting instrumental variables. This adjustment may elevate the likelihood of infringing upon the strong instrument assumption inherent to MR methodology. Nonetheless, we have mitigated this concern by filtering out weak instrumental variables utilizing *F*‐statistics (with *F* > 10). Lastly, the proportion of mediation attributable to inflammatory proteins appears to be relatively modest when assessing mediation effects, indicating the possible presence of other unidentified mediators. Furthermore, our findings remain theoretical at this point and necessitate validation through clinical or animal studies. Thus, additional research, encompassing both cellular and animal investigations, is essential to clarify these underlying mechanisms.

## 5 Conclusion

This research provides a comprehensive evaluation of the causal relationships between immune cell types, circulating inflammatory markers, and the onset of delirium. We have identified a pair of immune cell types that are causally linked to delirium, mediated by two distinct inflammatory proteins. Our findings highlight the importance of understanding the underlying mechanisms that connect immune cells, inflammatory proteins, and the occurrence of delirium. This suggests that future therapeutic approaches may focus on modulating immune cell levels by targeting the inflammatory microenvironment, thereby enhancing preventive strategies and facilitating the identification of at‐risk populations for delirium, enabling more effective targeted screening and management.

## Author Contributions

**Tongshuai Liu**: conceptualization, writing‐original draft, supervision, investigation, software, and visualization. **Xia Bi**: writing – original draft, formal analysis, and project administration. **Fuguo Ma**: writing – original draft, data curation, methodology, and resources. **Yanlin Chen**: conceptualization, writing – original draft, writing – review and editing, methodology, supervision, funding acquisition, and validation.

## Funding

This study was supported by the Qingdao Key Medical and Health Discipline Project.

## Ethics Statement

The present MR analysis was based on summary data from previous studies obtained from public databases that had gained relevant, informed consent and ethics approval. No ethical permit is required for the secondary analysis of summary data.

## Consent

The authors have nothing to report.

## Conflicts of Interest

The authors declare no conflicts of interest.

## Peer Review

The peer review history for this article is available at https://publons.com/publon/10.1002/brb3.70811.

## Supporting information



Supplementary Tables

Supplementary Tables

## Data Availability

The data that support the findings of this study are available from the corresponding author upon reasonable request.
